# Effects of self-control of feedback timing on motor learning

**DOI:** 10.3389/fpsyg.2025.1638827

**Published:** 2025-09-19

**Authors:** Kazunori Akizuki, Kosuke Takeuchi, Jun Yabuki, Kazuto Yamaguchi, Ryohei Yamamoto, Tatsuya Kaneno

**Affiliations:** ^1^Department of Physical Therapy, Mejiro University, Saitama, Japan; ^2^Department of Physical Therapy, Kobe International University, Kobe, Japan; ^3^Department of Rehabilitation, Nihon Institute of Medical Science, Saitama, Japan; ^4^Department of Rehabilitation, Kumamoto Health Science University, Kumamoto, Japan; ^5^Department of Occupational Therapy, Graduate School of Human Health Sciences, Tokyo Metropolitan University, Tokyo, Japan

**Keywords:** motor learning, self-control, visual feedback, feedback timing, concurrent feedback, terminal feedback, intrinsic motivation, information processing

## Abstract

**Introduction:**

Although the effects of self-control on motor learning have been well studied, the effects of self-controlled feedback timing have not been thoroughly investigated. Therefore, this study aimed to examine the effects of self-controlled feedback timing on motor learning through two experiments.

**Methods:**

In Experiment 1, participants were randomly assigned to one of three groups: concurrent feedback, terminal feedback, or self-controlled feedback. The procedure included a pre-test, practice session, and a retention test conducted 1 week after the practice, with visual feedback provided only during the practice session. Participants also completed three subscales of the Intrinsic Motivation Inventory before/after the practice session and the NASA-Task Load Index (NASA-TLX) after the practice session. In Experiment 2, participants were randomly assigned to either a self-controlled feedback group or a yoked feedback group, following the same procedure as in Experiment 1.

**Results:**

In Experiment 1, the concurrent feedback group demonstrated the smallest performance errors during practice, while the terminal feedback group showed the largest performance errors. However, both the self-controlled and terminal feedback groups exhibited significantly smaller errors than the concurrent feedback group in the retention test. In Experiment 2, the self-controlled group made significantly fewer errors than the yoked group in the retention test. The self-controlled group showed significantly higher intrinsic motivation and significantly lower scores in the performance subscale of NASA-TLX than the yoked group.

**Conclusion:**

In the present study, concurrent visual feedback interfered with motor learning by inducing a dependency on visual feedback. Our findings suggest that self-controlled feedback timing may overcome the potential negative effects of concurrent visual feedback through the positive influence of self-control, which may arise through the involvement of both intrinsic motivation and information processing.

## 1 Introduction

Motor learning is a critical process in sports and rehabilitation. In sports, it contributes to enhanced athletic performance (Benjaminse et al., 2017; Goudini et al., 2019; van der Meer et al., 2024) and injury prevention (Benjaminse et al., 2015; Gokeler et al., 2018). In rehabilitation, motor learning helps improve activities of daily living (Charlton et al., 2021; Levin and Demers, 2021) and enhance quality of life (Dettmers et al., 2005; Maier et al., 2019). Consequently, there has been growing interest in multifaceted investigations into the mechanisms of motor learning and the development of strategies to optimize it (Akizuki et al., 2022; Czyz et al., 2024; Leech et al., 2022; Nicklas et al., 2024; Spampinato and Celnik, 2021).

Feedback is a key factor influencing motor learning (Bugnon et al., 2023). Feedback refers to information available during or after practice that pertains to the outcome of a movement. It can be categorized as either intrinsic (derived from sources such as visual and proprioceptive inputs) or extrinsic (provided by an external source; e.g., coach, therapist, teacher, or device) feedback and is known as augmented feedback (Schmidt and Lee, 2019). Previous studies have shown that the frequency and timing of augmented feedback affect motor learning (Akizuki et al., 2019; Hebert and Coker, 2021; Liu et al., 2023; Sattelmayer et al., 2016; Sigrist et al., 2013a). For example, concurrent feedback delivered during task execution enables real-time error correction based on augmented feedback, which is generally considered to enhance task performance during practice more effectively than terminal feedback provided after task completion. However, concurrent feedback has also been associated with overreliance on augmented feedback (Annett, 1959; Salmoni et al., 1984), leading to performance deterioration during a retention test where such feedback is no longer available (Schmidt and Wulf, 1997; Vander Linden et al., 1993; Winstein et al., 1996).

The effects of concurrent feedback have been shown to depend on task complexity and learners’ skill level (Guadagnoli and Lee, 2004; Wulf and Shea, 2002). Sigrist et al. (2015) reported that concurrent visual feedback facilitated the learning of a motor task using a realistic rowing simulator, demonstrating the effectiveness of concurrent visual feedback in complex tasks. Yamamoto et al. (2019) investigated the influence of feedback timing on a weight-shifting task and found that concurrent feedback was particularly beneficial for individuals with lower skill levels. Yamamoto et al. (2022) conducted an experiment using a grasping force control task in community-dwelling older adults and found that concurrent visual feedback promoted motor learning. One potential reason why concurrent feedback is effective under conditions of high task complexity or low skill level is its ability to reduce cognitive load (Sigrist et al., 2013a; Wulf and Shea, 2002). Compared with terminal feedback, concurrent feedback may provide stronger guidance toward correct movement patterns, making it easier for learners to understand even complex task structures (Huegel and O’Malley, 2010; Wolpert and Flanagan, 2010). Consequently, the cognitive benefits of reducing mental workload may outweigh the disadvantages associated with increased feedback dependency and facilitate motor learning (Akizuki and Ohashi, 2015). However, because the effect of feedback timing varies depending on the complexity of the task and the learner’s skill level, there are limitations to generalizing the findings obtained under specific conditions (Guadagnoli and Lee, 2004).

One potential approach to flexibly harnessing the benefits of feedback timing without being constrained by specific task conditions is to allow learners to control the feedback’s timing. The effectiveness of “self-controlled” practice, in which learners are given autonomy over certain aspects of the task or practice conditions, has been demonstrated in various tasks and learning contexts (Hebert and Coker, 2021; Iwatsuki and Otten, 2021; Janelle et al., 1995; Sanli et al., 2013; van der Meer et al., 2024). However, there is no consensus regarding the mechanisms underlying its effectiveness. Lewthwaite et al. (2015) reported that allowing learners to choose the color of the golf ball in a putting task, the preferences for paintings in a laboratory setting, or subsequent tasks after a balance exercise improved practice performance and retention test. As these choices were unrelated to task performance, the authors concluded that the benefits of self-controlled practice were attributable to the act of choosing itself. They further argued that the act of choice satisfies the basic psychological need for autonomy (Deci and Ryan, 2000, 2008), thereby enhancing intrinsic motivation and promoting motor performance and learning (Chiviacowsky, 2014; Wulf and Lewthwaite, 2016).

In contrast, Carter and Ste-Marie (2017a) investigated the effects of task-relevant choices (e.g., feedback schedule), task-irrelevant choices (e.g., post-experiment activity type and arm-wrap color), and no-choice conditions on motor performance and learning using an elbow extension or flexion task guided by a target waveform. Their findings indicated that motor learning was enhanced only in the task-relevant choice condition. Performance and learning outcomes in the task-irrelevant choice condition were comparable to those in the no-choice condition. Moreover, although perceived competence and autonomy did not differ between choice conditions, participants in the task-relevant choice group exhibited more accurate error estimations. These results suggest that choice may facilitate motor learning by promoting information-processing activities such as error estimation (Carter and Ste-Marie, 2017b; Grand et al., 2015). Although a unified account of the mechanisms underlying how self-control facilitates motor learning is yet to be established, some have questioned the robustness of the beneficial effects of self-control (e.g., McKay et al., 2025; St. Germain et al., 2023). Nevertheless, allowing learners to self-control practice parameters in contexts where no clear guidelines exist for optimal conditions may be a reasonable and effective strategy for enhancing learning. However, the specific effects of self-controlled feedback timing have not yet been examined empirically.

Therefore, the present study aimed to examine the effects of self-controlled feedback timing using a grasping force control task (Yabuki et al., 2025; Yamamoto et al., 2022). This investigation will clarify the role of feedback timing in motor learning in the context of grasping force control and determine whether self-controlled feedback timing facilitates motor learning. Furthermore, by assessing participants’ intrinsic motivation and mental workload during task execution, this study will contribute to a deeper understanding of the mechanisms underlying the effects of self-controlled practice.

## 2 Materials and methods

### 2.1 Experimental design

**Figure 1:**
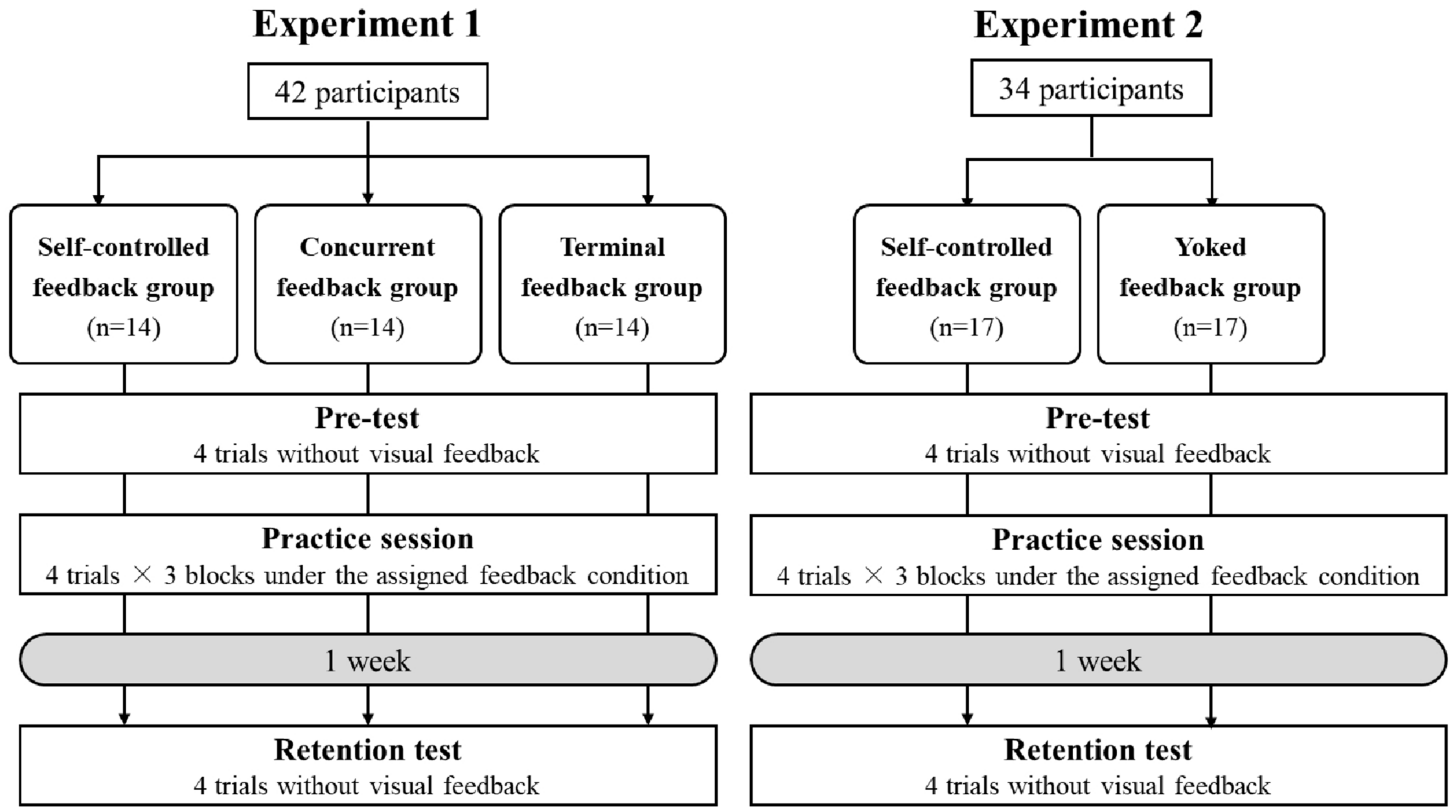
Experimental designs for Experiments 1 (left) and 2 (right). Experiment 1 aimed to investigate the effect of self-controlled feedback timing on motor learning in comparison with concurrent and terminal feedback conditions. Experiment 2 aimed to clarify the true effect of self-controlled feedback timing. The same experimental task (adjusting the grasping force task) and procedure were used in Experiments 1 and 2, with different feedback conditions.

We conducted two experiments to examine the effects of self-control of feedback timing on motor learning in a grasping force control task. In Experiment 1, we compared the self-controlled feedback group with the concurrent and terminal feedback groups to investigate whether self-control of feedback timing is beneficial for motor learning compared with traditional feedback timing (Figure 1, left). In Experiment 2, we compared the self-controlled and yoked feedback groups to clarify the true effect of self-controlled feedback timing. Participants in both groups in Experiment 2 executed the experimental task under the same conditions, except for the presence of self-control (Figure 1, right). The same motor task was used in Experiments 1 and 2, and the experimental procedure in all groups was standardized, except for the feedback timing condition.

### 2.2 Participants

Power analyses were conducted to determine the required sample size using G*Power 3.1 (Heinrich Heine University, Düsseldorf, Germany). A repeated-measures analysis of variance (ANOVA) with a within-between interaction was specified, assuming a medium effect size (Cohen’s *f* = 0.25), an *α* error level probability of 0.05, and a statistical power of 0.80 (1-β error probability; Serdar et al., 2021). The effect size specification followed the default settings of GPower 3.0. The analysis revealed that minimum total sample sizes of 42 and 34 were required for Experiments 1 and 2, respectively. Therefore, 42 (*M*_age_ = 21, SD = 1.2; 13 women and 29 men) and 34 participants (*M*_age_ = 20.3, SD = 0.73; eight women and 26 men) were recruited for Experiments 1 and 2, respectively.

All participants were right-handed, as determined by the Edinburgh Handedness Inventory. None of the participants reported any neurological or orthopedic condition before participating in the study. Participants had no prior experience with the experimental task and were not informed of the specific purpose of our study. A preliminary explanation of the study details was provided to all participants and written consent was obtained. The study protocol was approved by the institutional review board of Kobe International University (approval number: G2019-090).

### 2.3 Task and apparatus

A device (iWakka, Nagoya Institute of Technology, Japan) was used to measure the grasping force. The device had a cylindrical shape with an 80 mm height and a 65 mm diameter. A plate spring was installed inside the cylindrical device; as it opened and closed, the plate spring was distorted. The degree of distortion was then measured with a gauge and analyzed by a computer to measure the change in the grasping force (in grams) over time (in seconds). In addition to being able to display the target line on the monitor, this device immediately displayed the measured value of the grasping force on the monitor during task performance, so that it is superimposed on the target line. After completing the task, the target line and time-course changes in the grasping force were superimposed and displayed on the monitor.

The participants were instructed to adjust their grasping force according to the target line displayed on the monitor. We configured the target line in three phases: the first-, second-, and third-phase target values were set to 100, 400, and 250 g, respectively. In addition, a preparation phase that did not display the target line on the monitor was set up prior to the first phase. Each phase was set at 10 s, and a metronome (6 bpm) was used to tell the participant to shift phases.

**Figure 2:**
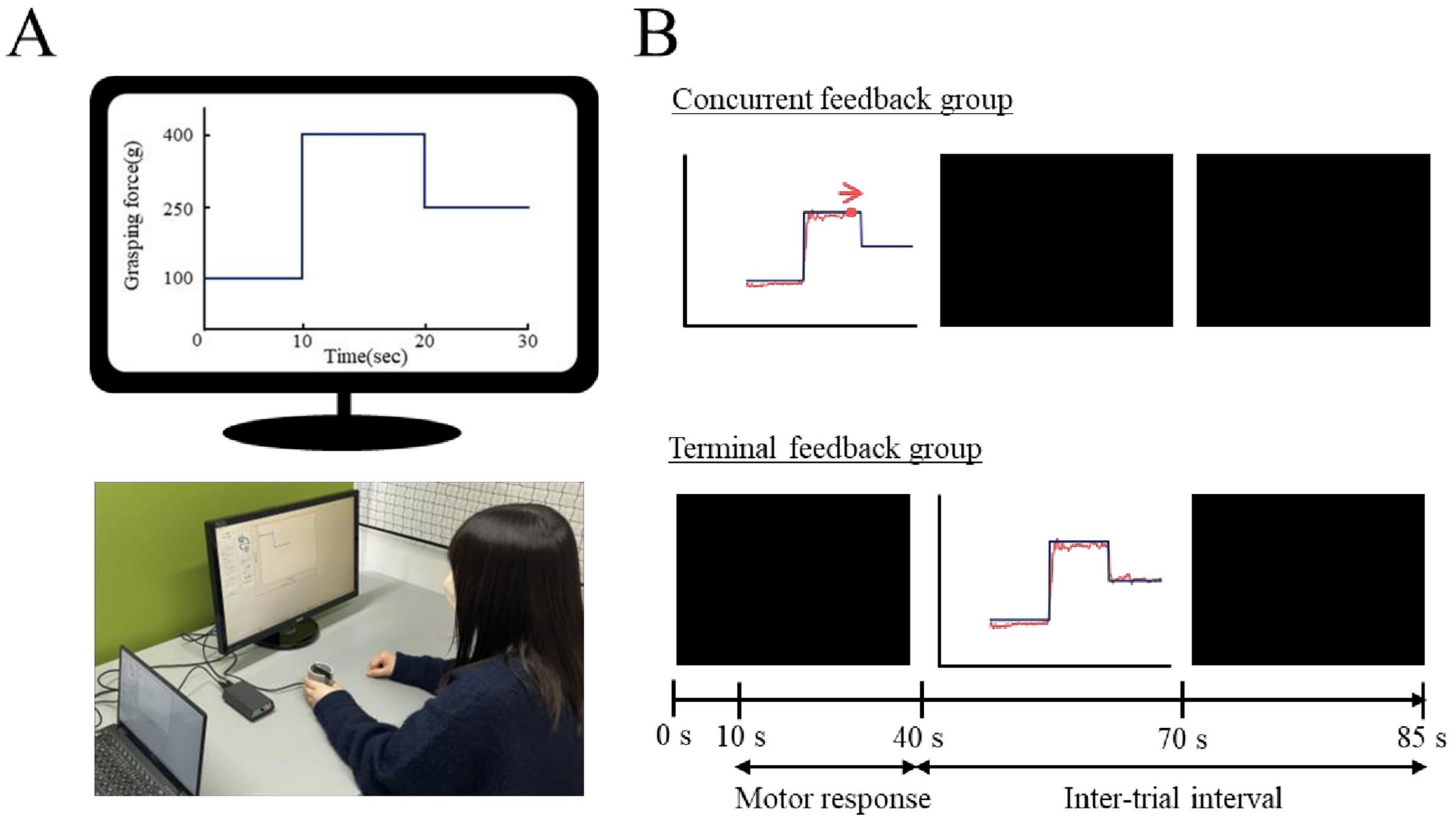
The grasping force task **(A)** and the time course of a trial in the concurrent and terminal feedback groups **(B)**. Participants in the self-controlled feedback group could choose between two feedback timings (concurrent or terminal) before each trial. The motor response started after the preparation phase (10 s). The duration when participants were exposed to augmented feedback per trial was 30 s in all groups.

To standardize the experimental conditions, we prepared an experimental environment based on a previous study (Kaneno et al., 2019). For example, the assessment was conducted in a quiet environment. The participants were seated with their legs shoulder-width apart, and their knee joints kept in a 90 ° flexed position. The distance from the table to the body was maintained at 10 cm, and the 17″ computer monitor was placed 50 cm from the edge of the table (Figure 2A).

### 2.4 Outcomes

#### 2.4.1 The root mean square error

The root mean square error (RMSE) from the absolute error per unit time between the target and measured values was calculated as the overall performance accuracy. To extract stable waveforms for each phase, only 3–8 s (first phase), 13–18 s (second phase), and 23–28 s (third phase) were used in the analysis. Through this manipulation, the influences of timing errors accompanying phase shifting were removed.

#### 2.4.2 Intrinsic motivation

Similar to previous studies (Abbas and North, 2018; Badami et al., 2011), a nine-item questionnaire consisting of the interest/enjoyment, perceived competence, and effort/importance sub-scales of the Intrinsic Motivation Inventory (IMI) (McAuley et al., 1989) was adapted for use in the study. This assessed participants’ subjective experiences in performing the experimental task. The items were scored on a seven-point Likert scale ranging from 1 (“strongly disagree”) to 7 (“strongly agree”).

#### 2.4.3 NASA-task load index

The NASA-Task Load Index (NASA-TLX) is a widely used psychometric tool for assessing subjective workload (Hart and Staveland, 1988). Twenty-step bipolar scales were used to obtain ratings for the six subscales: mental demand, physical demand, temporal demand, performance, effort, and frustration. Mental, physical, and temporal demands were categorized as task-related scales, while performance and effort were behavior-related scales, and frustration was classified as a subjective scale. A score ranging from 0 to 100 (assigned to the nearest five points) was obtained for each scale. The NASA-TLX has demonstrated broad applicability across domains such as aviation, human–machine interaction, and healthcare (Hart, 2006), with its reliability and validity well established in the literature (Devos et al., 2020; Hart and Staveland, 1988).

### 2.5 Experimental procedures

#### 2.5.1 Experiment 1

Three experimental groups (n = 14 per group) were created: concurrent, terminal, and self-controlled feedback. During the practice session, participants in the concurrent feedback group were allowed to watch the monitor displaying both the target line and the measured grasping force value. The participants were instructed to adjust their grasping force to trace the target line as accurately as possible. The target line and measured grasping force values were blinded immediately after each trial was completed. Participants in the terminal feedback group were instructed to watch the monitor while performing the task. However, the monitor did not display the target line or the measured grasping force because the screen of the monitor was blinded to the experimental manipulation. After each practice trial, the participants could see the target line and the line created by the time-course changes in the grasping force. These lines were displayed on the monitor as augmented feedback for 30 s after the completion of the trial. This augmented feedback duration was determined to be the same as that in the concurrent feedback group. Finally, while participants in the self-controlled feedback group received feedback on every trial during the practice session, they could choose between two feedback timings (concurrent or terminal feedback) before each trial (Figure 2B).

The participants visited the laboratory twice on separate days, with an interval of 1 week between visits. On the first visit, all participants received an explanation of the task and experimental schedule. During this explanation, the target line and phase-specific target values were visually presented to all the participants (Figure 2A). They were instructed to adjust their grasping force in time with a metronome. In addition, participants were informed that they would take a retention test 1 week later under the same conditions as the pre-test (i.e., without augmented feedback). Following the explanation, the participants completed a familiarization trial without augmented feedback but with target values cued by the experimenter in time with a metronome to ensure comprehension of both the performance goal and the experimental procedure. Subsequently, they performed a pre-test and a practice session. The pre-test consisted of four trials without augmented feedback. Immediately after completing the pre-test, the IMI was administered. The practice session, which consisted of three blocks of four trials, started 3 min after the pre-test ended. During the practice session, participants practiced the experimental task under the assigned feedback condition. Trials were 40 s in duration, with 45 s inter-trial intervals, and a 3 min rest period after each block. After the practice session was completed, the IMI and the NASA-TLX were administered. On the second visit, the participants performed a warm-up trial without augmented feedback in advance and then completed four trials as a retention test.

#### 2.5.2 Experiment 2

Two experimental groups (*n* = 17 per group) were created: self-controlled and yoked feedback. The feedback condition of self-controlled feedback was the same as that in Experiment 1. Each participant in the yoked feedback group was matched with a participant in the self-controlled group. The yoked feedback group participants were told that the experimenter had made a schedule of feedback timing in advance. Participants in the yoked feedback group matched the schedules chosen by their counterparts in the self-controlled group. Therefore, the presence or absence of choice was a singular difference between the yoked and self-controlled feedback groups. Consequently, the effects of feedback timing were controlled. Although the experimental conditions for comparison with the self-controlled feedback group were different, the experimental procedure was the same as in Experiment 1.

### 2.6 Data analysis

For Experiment 1, the RMSE was analyzed using a 3 (group: self-controlled, concurrent, terminal) × 3 (block) mixed-model ANOVA with repeated measures on the last factor for the practice session, and a 3 (group) × 2 (test: pre-test, retention test) ANOVA with repeated measures on the last factor for the test session. Each of the three sub-scale scores of the IMI before and after practice were analyzed using a 3 (group) × 2 (timing: before practice, after practice) ANOVA with repeated measures on the last factor, as each subscale reflects a distinct aspect of intrinsic motivation. The NASA-TLX subscales were analyzed using multivariate ANOVA (MANOVA) with group as the independent variable, given that the subscales are conceptually related and jointly represent the construct of workload. This multivariate approach enabled us to detect overall patterns across workload dimensions, while still allowing examination of individual subscales if necessary.

For Experiment 2, the RMSE was analyzed using a 2 (group: self-controlled, yoked) × 3 (block) mixed-model ANOVA with repeated measures on the last factor for the practice session, and a 2 (group) × 2 (test: pre-test, retention test) ANOVA with repeated measures on the last factor for the test session. Each of the three sub-scale scores of the IMI and the NASA-TLX were analyzed using the same methods as in Experiment 1.

IBM SPSS Statistics version 29 (IBM Corp., Armonk, NY, United States) was used for statistical analyses. In all analyses, a significance level of *p* < 0.05 was used. Post-hoc comparisons were conducted using Bonferroni’s test, where appropriate.

## 3 Results

### 3.1 Experiment 1

#### 3.1.1 RMSE

Participants in the self-controlled feedback group chose concurrent feedback, with an average of 60.7, 44.6, and 28.6% trials in Blocks 1, 2, and 3, respectively. A one-way repeated-measures ANOVA with block as the within-subject factor revealed a significant main effect of block [*F*(2, 26) = 8.47, *p* = 0.001, *η_p_^2^* = 0.394]. Bonferroni-corrected multiple comparisons indicated significant differences between Block 1 and Block 2 (*p* = 0.040) and between Block 1 and Block 3 (*p* = 0.007), whereas no significant difference was found between Block 2 and Block 3 (*p* = 0.285). Thus, participants in the self-controlled feedback group showed a significant shift from choosing concurrent feedback to choosing terminal feedback.

**Figure 3:**
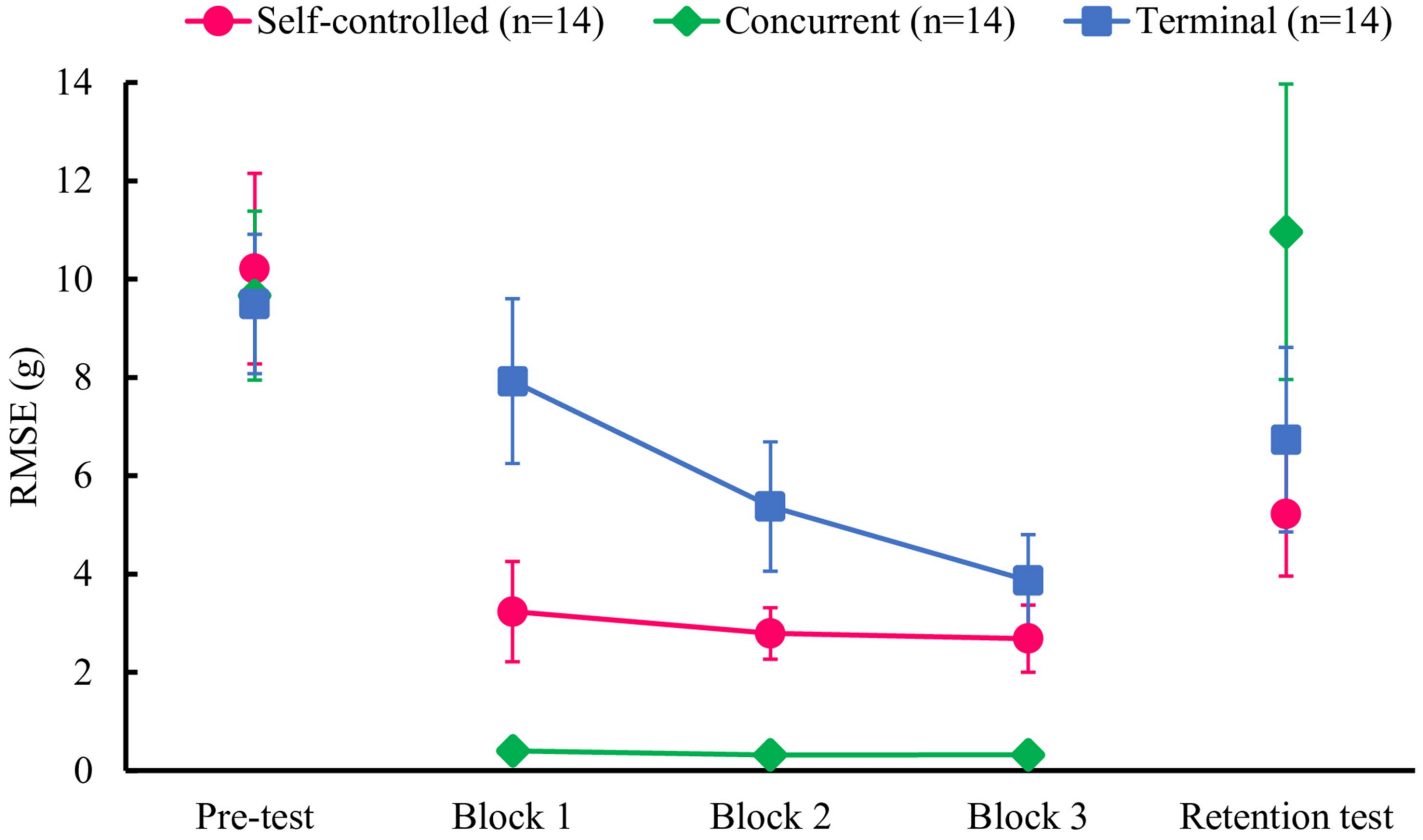
Mean RMSEs for the practice session (Blocks 1–3) and tests (pre, retention) in Experiment 1. There were no significant differences among the three groups in the pre-test. On the retention test, the terminal and the self-controlled feedback groups showed significantly greater accuracy than the concurrent feedback group, whereas the difference between the terminal and the self-controlled feedback groups was not significant. Error bars represent 95% confidence intervals.

According to the ANOVA results for practice sessions, the main effects of group [*F*(2, 39) = 55.73, *p* < 0.001, *η_p_^2^* = 0.741] and block [*F*(2, 78) = 16.05, *p* < 0.001, *η_p_^2^* = 0.292], and the group × block interaction [*F*(4, 78) = 10.18, *p* < 0.001, *η_p_^2^* = 0.343] were significant. The post-hoc analysis for the group × block interaction indicated that the concurrent feedback group performed significantly better than the self-controlled and terminal feedback groups in all blocks, and the self-controlled feedback group had significantly higher accuracy than the terminal feedback group in Block 1 and Block 2 (Block 1: *p* < 0.001; Block 2: *p* < 0.001; Block 3: *p* = 0.060). Furthermore, the terminal feedback group showed significant improvement in accuracy during the practice session. This was confirmed by multiple comparisons of blocks in the terminal feedback group (*p_s_* < 0.001 for all comparisons). However, no significant differences were observed between the blocks in the concurrent and self-controlled feedback groups (Figure 3).

On the test session, the ANOVA results revealed that the main effect of group [*F*(2, 39) = 4.55, *p* = 0.017, *η_p_^2^* = 0.189] and test [*F*(1, 39) = 6.18, *p* = 0.017, *η_p_^2^* = 0.137], and the group × test interaction [*F*(2, 39) = 4.53, *p* = 0.017, *η_p_^2^* = 0.189], were significant. The post-hoc analysis for the group × test interaction indicated that the terminal (*M* = 6.7, *SD* = 3.6) and the self-controlled (*M* = 5.2, *SD* = 2.4) feedback groups showed more accurate performance than the concurrent feedback group (*M* = 11.0, *SD* = 5.7) in the retention test (*p* = 0.031 and *p* = 0.002, respectively), whereas there was no significant difference between the terminal and the self-controlled feedback groups. In the pre-test, no significant differences were observed between the groups. Moreover, a significant improvement from pre- to retention test was found in the self-controlled feedback group (*p* = 0.002), whereas no significant improvements were found in the terminal (*p* = 0.073) or the concurrent (*p* = 0.392) feedback groups.

#### 3.1.2 IMI

The ANOVA for interest/enjoyment revealed a significant main effect of timing [*F*(1, 39) = 45.33, *p* < 0.001, *η*_p_^2^ = 0.538]. In contrast, the main effects of group [*F*(2, 39) = 1.01, *p* = 0.374, *η_p_^2^* = 0.049] and group × timing interaction [*F*(2, 39) = 2.20, *p* = 0.124, *η_p_^2^* = 0.101] were not significant. The ANOVA for perceived competence revealed a significant main effect of timing [*F*(1, 39) = 66.82, *p* < 0.001, *η*_p_^2^ = 0.631]. In contrast, the main effects of group [*F*(2, 39) = 0.37, *p* = 0.694, *η_p_^2^* = 0.019] and group × timing interaction [*F*(2, 39) = 0.74, *p* = 0.483, *η_p_^2^* = 0.037] were not significant. The ANOVA for effort/importance revealed a significant main effect of timing [*F*(1, 39) = 12.39, *p* = 0.001, *η*_p_^2^ = 0.241]. In contrast, the main effects of group [*F*(2, 39) = 1.63, *p* = 0.209, *η_p_^2^* = 0.077] and group × timing interaction [*F*(2, 39) = 0.58, *p* = 0.566, *η_p_^2^* = 0.029] were not significant (Table 1).

**Table 1:** The intrinsic motivation inventory sub-scale scores before and after practice by feedback condition in Experiments 1 and 2.

Measures	Exp. 1/groups			Exp. 2/groups	
	Self-controlled	Concurrent	Terminal	Self-controlled	Yoked
Interest/Enjoyment					
Before	5.50 (1.04)	4.67 (1.85)	5.33 (1.11)	5.76 (1.11)	5.27 (0.99)
After	6.55 (0.61)	6.40 (0.66)	6.19 (0.68)	6.63 (0.58)	6.14 (0.68)
Perceived competence					
Before	3.02 (0.82)	2.98 (1.16)	3.00 (1.41)	3.10 (0.83)	3.06 (0.96)
After	4.81 (1.20)	4.38 (1.20)	4.26 (0.74)	4.75 (1.10)	3.86 (1.30)
Effort/Importance					
Before	6.02 (0.71)	6.10 (0.89)	5.83 (0.64)	6.04 (0.64)	5.98 (0.51)
After	6.50 (0.62)	6.52 (0.45)	6.05 (0.70)	6.45 (0.58)	6.24 (0.50)

The range of each subscale of the Intrinsic Motivation Inventory is 0–7 points. Standard deviations are in parentheses.

#### 3.1.3 NASA-TLX

The MANOVA with NASA-TLX subscales as the dependent variables revealed a significant multivariate main effect for group [*F*(12, 70) = 3.36, *p* < 0.001, Pillai’s Trace = 0.731]. Follow-up univariate ANOVAs indicated significant group effects only in the performance [*F*(2, 39) = 7.35, *p* = 0.002, *η_p_^2^* = 0.274] and frustration [*F*(2, 39) = 5.83, *p* = 0.006, *η_p_^2^* = 0.230] subscales. Post-hoc comparisons for the performance subscale revealed that the terminal feedback group had a higher score than the self-controlled feedback group (*p* = 0.002). Notably, a high score on the performance dimension of the NASA-TLX indicates poor perceived performance. The post-hoc analysis for the frustration subscale revealed that the terminal and self-controlled feedback groups showed higher scores than the concurrent feedback group (*p* = 0.007 and *p* = 0.048, respectively) (Table 2).

**Table 2:** The NASA-TLX subscale scores by feedback condition in Experiments 1 and 2.

Measures	Exp. 1/groups			Exp. 2/groups	
	Self-controlled	Concurrent	Terminal	Self-controlled	Yoked
Mental demand	69.3 (27.4)	77.9 (25.0)	85.7 (15.7)	72.6 (26.1)	69.7 (22.0)
Physical demand	36.1 (23.9)	26.4 (25.1)	41.4 (32.2)	35.6 (22.4)	31.8 (26.5)
Temporal demand	30.4 (27.1)	13.6 (17.5)	33.9 (29.0)	33.5 (26.1)	19.1 (18.9)
Performance	30.7 (16.0)	43.2 (30.1)	65.7 (25.2)	31.8 (16.7)	56.8 (20.8)
Effort	88.6 (14.2)	83.9 (17.7)	83.6 (15.4)	88.5 (13.6)	84.7 (12.7)
Frustration	35.7 (31.1)	10.0 (13.4)	43.2 (32.2)	30.0 (30.9)	21.2 (22.9)

The range of each subscale of the NASA-Task Load Index (NASA-TLX) is 0–100 points. Standard deviations are in parentheses.

### 3.2 Experiment 2

#### 3.2.1 RMSE

Participants in the self-controlled feedback group chose concurrent feedback, with an average of 58.8, 45.6, and 27.9% trials in Blocks 1, 2, and 3, respectively. A one-way repeated-measures ANOVA with block as the within-subject factor revealed a significant main effect of block [*F*(2, 32) = 10.66, *p* < 0.001, *η_p_^2^* = 0.400]. Bonferroni-corrected multiple comparisons indicated significant differences between Block 1 and Block 2 (*p* = 0.045) and between Block 1 and Block 3 (*p* = 0.002), whereas no significant difference was found between Block 2 and Block 3 (*p* = 0.105).

**Figure 4:**
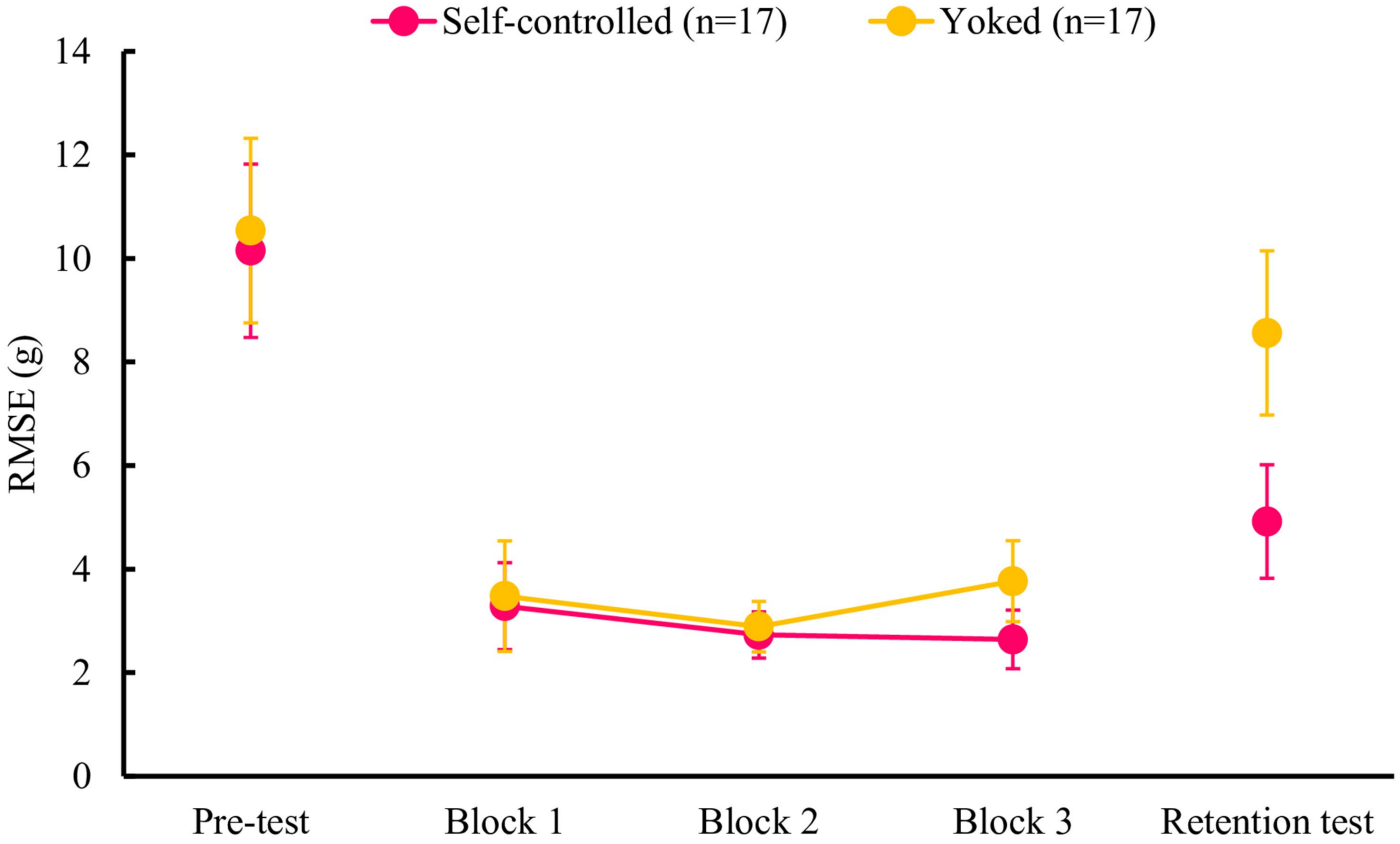
Mean RMSEs for the practice session (Blocks 1–3) and tests (pre, retention) in Experiment 2. There were no significant differences between the groups in the pre-test and practice sessions. On the retention test, the self-controlled feedback group demonstrated significantly greater accuracy than the yoked feedback group. Error bars represent 95% confidence intervals.

Based on the results of the ANOVA for practice sessions, the main effect of group [*F*(1, 32) = 2.84, *p* = 0.102, *η_p_^2^* = 0.081] and block [*F*(2, 64) = 1.18, *p* = 0.313, *η_p_^2^* = 0.036], and the group × block interaction [*F*(2, 64) = 1.03, *p* = 0.362, *η_p_^2^* = 0.031] were not significant (Figure 4).

On the test session, the ANOVA results revealed that the main effect of group [*F*(1, 32) = 5.49, *p* = 0.026, *η_p_^2^* = 0.146] and test [*F*(1, 32) = 24.97, *p* < 0.001, *η_p_^2^* = 0.438], and the group × test interaction [*F*(1, 32) = 5.08, *p* = 0.031, *η_p_^2^* = 0.137], were significant. The post-hoc analysis for the group × test interaction indicated that the self-controlled feedback group (*M* = 4.9, *SD* = 2.3) was superior to the yoked feedback group (*M* = 8.6, *SD* = 3.3) in the retention test (*p* < 0.001), whereas no significant differences were observed between groups in the pre-test (*p* = 0.756). Moreover, a significant improvement from pre- to retention test was found in the self-controlled feedback group (*p* < 0.001) but not in the yoked feedback group (*p* = 0.061).

#### 3.2.2 IMI

The ANOVA for interest/enjoyment results revealed that the main effect of timing was significant [*F*(1, 32) = 26.00, *p* < 0.001, *η_p_^2^* = 0.448]. In contrast, the main effects of group [*F*(1, 32) = 4.03, *p* = 0.053, *η_p_^2^* = 0.112] and group × timing interaction [*F*(1, 32) = 0.00, *p* = 1.000, *η_p_^2^* = 0.000] were not significant. The ANOVA for perceived competence results revealed that the main effect of timing [*F*(1, 32) = 37.23, *p* < 0.001, *η_p_^2^* = 0.538] and the group × timing interaction [*F*(1, 32) = 4.41, *p* = 0.044, *η_p_^2^* = 0.121] were significant. However, the main effect of group [*F*(1, 32) = 2.29, *p* = 0.140, *η_p_^2^* = 0.067] was not significant. The post-hoc analysis for the group × timing interaction indicated that participants in the self-controlled feedback group reported higher perceived competence after practice than did participants in the yoked feedback group (*p* = 0.041), although there was no significant difference between groups before practice (*p* = 0.900). Furthermore, a significant increase in perceived competence from before to after practice was observed in both feedback groups (self-controlled group: *p* < 0.001, yoked group: *p* = 0.008). The ANOVA for effort/importance results revealed that the main effect of timing was significant [*F*(1, 32) = 13.25, *p* < 0.001, *η_p_^2^* = 0.293]. In contrast, the main effects of group [*F*(1, 32) = 0.66, *p* = 0.421, *η_p_^2^* = 0.020] and group × timing interaction [*F*(1, 32) = 0.73, *p* = 0.398, *η_p_^2^* = 0.022] were not significant (Table 1).

#### 3.2.3 NASA-TLX

The MANOVA revealed a significant multivariate main effect for group [*F*(6, 27) = 2.80, *p* = 0.030, Hotelling’s Trace = 0.622]. Follow-up univariate ANOVAs indicated a significant effect only for the performance subscale [*F*(1, 32) = 14.92, *p* < 0.001, *η_p_^2^* = 0.318]. *Post hoc* comparisons revealed that the self-controlled feedback group had a significantly lower NASA-TLX performance score than the yoked feedback group (*p* < 0.001). In other words, participants in the self-controlled feedback group reported higher perceived performance than those in the yoked feedback group. However, no group differences were found in scores of the other NASA-TLX dimensions (Table 2).

## 4 Discussion

This study investigated the effects of self-controlled feedback timing through two experiments. In Experiment 1, the impact of self-controlled feedback timing on motor learning during a grasping force control task was examined by comparing it with concurrent and terminal feedback conditions. In Experiment 2, to isolate the effect of self-control in the self-controlled condition, a yoked group—matched in all aspects except for the ability to choose feedback timing—was employed.

In Experiment 1, although concurrent feedback resulted in superior performance during practice compared to terminal feedback, participants in the terminal feedback group outperformed those in the concurrent feedback group on a retention test conducted 1 week later. This finding is consistent with those of previous studies (Ferris et al., 2022; Sigrist et al., 2013b; Walsh et al., 2009; Xeroulis et al., 2007), suggesting that terminal feedback is more effective for motor learning in the grasping force control task employed in the present study (Yabuki et al., 2025). This result can be interpreted from the perspectives of the guidance hypothesis (Marco-Ahulló et al., 2024; Salmoni et al., 1984) and visual feedback dominance (Hecht and Reiner, 2009). According to the guidance hypothesis, participants receiving concurrent feedback likely benefited from real-time error information that facilitated immediate movement corrections, resulting in fewer performance errors during practice compared with the other groups. However, because their movement control strategies relied on augmented feedback, the removal of such feedback during the retention test may have led to a decline in performance. In contrast, participants in the terminal feedback group did not receive real-time error information and, therefore, were required to engage in internal processing, such as matching proprioceptive cues experienced during the trial with augmented feedback provided afterward, to improve their performance in subsequent trials. Such internal processing is thought to promote motor learning (Anderson et al., 2005; Hodges and Lohse, 2020; Lee et al., 1994). Moreover, concurrent visual feedback may dominate processing when multiple sensory feedback modalities are available, potentially interfering with the integration of other sensory information (Henriques and Cressman, 2012). Hecht and Reiner (2009) demonstrated the visual dominance effect, wherein visual input is processed preferentially over other modalities such as haptic or auditory input. Similarly, in a study of individuals with chronic stroke, Pellegrino et al. (2017) found that reliance on concurrent visual feedback during sitting balance training diminished the learning benefits of training. In the present study, the presence of concurrent visual feedback during task execution may have induced participants in the concurrent feedback group to adopt visually dominant information processing, which could have impaired their performance during the retention test, where visual feedback was no longer available (Goodwin and Goggin, 2018).

Furthermore, participants in the self-controlled group, despite frequently choosing concurrent feedback, particularly during the early phase of practice, demonstrated superior performance during practice compared to the terminal feedback group, and their retention test performance was similar to that of the terminal group. These findings suggest that self-control of feedback timing in a grasping force control task may counteract the negative effects of concurrent feedback or even provide benefits that outweigh those negative effects. However, the results of Experiment 1 did not allow us to determine whether the observed effect stemmed from the act of self-control of feedback timing or from the combined use of two different feedback timings. To isolate the contribution of self-control of feedback timing, Experiment 2 was conducted, wherein the self-controlled group was compared to the yoked group. The self-controlled group demonstrated superior motor learning compared with the yoked group. Given that the only difference between the two groups was the presence or absence of choice, the enhanced learning observed in the self-controlled group can be attributed to the opportunity to choose feedback timing.

Although several studies have reported the positive effects of self-control on motor learning (Aikin et al., 2020; Goudini et al., 2019; Hebert and Coker, 2021; Jimenez-Diaz et al., 2021; van der Meer et al., 2024), others have reported null effects (Bacelar et al., 2022; St. Germain et al., 2022; Yamamoto et al., 2025; Yantha et al., 2022) or only small effect sizes, even when benefits were found (McKay et al., 2022; McKay and Ste-Marie, 2020). Our findings support the positive effects of self-control. To the best of our knowledge, this is the first study to demonstrate that self-controlled feedback timing facilitates motor learning. The comparative results of the concurrent and terminal feedback groups in Experiment 1 suggest that choosing more terminal feedback was advantageous for motor learning in the experiment task. Of note, participants in the self-controlled group, despite not being informed in advance about the relative effectiveness of each feedback timing, spontaneously preferred concurrent feedback in the early phase of practice and transitioned to terminal feedback in the later phase. Such a shift in feedback timing appears to be a rational adjustment based on changes in skill levels and functional task difficulty (Guadagnoli and Lee, 2004; Sigrist et al., 2013a). It is plausible that participants initially favored concurrent feedback to better understand the structure of the task and later preferred terminal feedback to promote motor control strategies that were more reflective of test conditions (i.e., performance without real-time visual guidance). In the present study, such a reasonable adjustment occurred spontaneously, without explicit instruction. Drews et al. (2024) argued that the benefits of self-control in motor learning stem not from the possibility of choosing, but from the consequences of the specific choices made. This study did not allow us to clarify whether such strategic choices were made consciously or unconsciously. However, in situations where effective feedback timing is unknown in advance, allowing the learner to choose it for themselves may be a rational strategy for motor learning.

The mechanisms through which self-control influences motor learning have been discussed from the perspectives of intrinsic motivation (Hebert and Coker, 2021; Iwatsuki and Otten, 2021; Lewthwaite et al., 2015), information-processing (Barros et al., 2019; Carter and Ste-Marie, 2017a; Woodard and Fairbrother, 2020), and their integration (Bacelar et al., 2022). In the present study, the self-controlled feedback group demonstrated significantly higher perceived competence on IMI than the yoked feedback group. Moreover, the self-controlled feedback group had a significantly lower NASA-TLX performance score than the yoked feedback group. The performance dimension of the NASA-TLX reflects the difference between the learner’s desired state and the perceived actual state, and it has been suggested that the higher the score, the more information processing is required to correct the error (Akizuki et al., 2025). Therefore, self-control of feedback timing may have facilitated motor learning not only by enhancing intrinsic motivation but also by inducing more efficient information processing.

We propose that the effects of self-control may not result from either motivation or information processing alone, but rather from the dynamic interplay between the two. For example, increased motivation may lead learners to engage more actively with a task, thereby enhancing the depth of information processing (Kim et al., 2019). Conversely, greater cognitive effort during practice may activate learners’ sense of challenge, which, in turn, could foster greater motivation (Sanli et al., 2013). Kim et al. (2019) investigated the neurophysiological effects of self-controlled feedback on motor learning by measuring EEG activity during practice. Their results indicated that participants in the self-controlled group processed task-relevant stimuli (e.g., larger post-stimulus P3 amplitudes) and feedback (e.g., larger and earlier post-feedback P3 amplitudes) more actively than those in the yoked group, who, in turn, exhibited larger post-response error positivity (Pe) amplitudes, suggesting a greater focus on processing response errors. Additionally, although the self-controlled group showed enhanced P3 amplitudes following task stimuli and feedback in the prefrontal, frontal, and central regions, activity in the parietal region was reduced compared with the yoked group. These patterns suggest that the self-controlled group processed the information in a more consciously controlled manner. Such evidence supports the notion that information processing during practice differs between self-controlled and yoked participants. Therefore, our results indicate that allowing learners to control the timing of feedback enhances the dynamic interplay between intrinsic motivation and information-processing, which facilitates motor learning.

This study had some limitations. First, although we examined the effects of self-controlled feedback timing using a grasping force control task with potential applicability in rehabilitation settings, it remains uncertain whether similar effects would be observed in other types of motor tasks. As the grasping force control task relies heavily on visual information (Yabuki et al., 2025; Yamamoto et al., 2022), it is particularly susceptible to the detrimental effects of concurrent visual feedback. Consequently, the present findings may not generalize to tasks in which concurrent feedback is more effective than terminal feedback, such as a realistic rowing simulator (Sigrist et al., 2015) or a weight-shifting task (Yamamoto et al., 2019). Future studies should investigate whether the effects of self-controlled feedback timing observed here generalize to motor tasks that rely less on visual information and in which concurrent feedback may be more beneficial (e.g., rowing simulations or weight-shifting tasks).

Second, the current study was conducted among healthy young adults, and it is uncertain whether the same results would be observed in populations of children, older adults, or individuals with clinical conditions. For example, previous research has shown that the effects of concurrent visual feedback differ between younger and older adults (Wishart et al., 2002). Additionally, as Drews et al. (2024) noted, the benefits of self-control in motor learning are determined by the choices made, suggesting that cognitive function or psychological state may influence how learners select feedback timing (Chiviacowsky et al., 2008; Kaefer et al., 2014).

Third, the basis on which participants in the self-controlled group chose concurrent or terminal feedback in this study remains unclear. Previous research on self-controlled feedback has shown that learners tend to request feedback after successful trials (Chiviacowsky and Wulf, 2002) and that the frequency of feedback under self-controlled conditions is not always optimally adjusted (Drews et al., 2024). In the current study, participants in the self-controlled group spontaneously shifted toward selecting more terminal feedback as practice progressed, a strategy that appears to facilitate motor learning (Guadagnoli and Lee, 2004; Sigrist et al., 2013a). However, this study could not explain why such a transition occurred. Future studies should aim to investigate the cues, whether consciously or unconsciously perceived, that guide learners’ choices regarding feedback timing. Such investigations would contribute to a more precise understanding of the mechanisms through which self-control influences motor learning.

Finally, the simplicity of the grasping force control task and the limited amount of practice provided to participants represent important limitations. In the present study, only the self-controlled feedback group demonstrated significant improvement from the pre-test to the retention test; however, this effect may have been influenced by task simplicity and restricted practice. Accordingly, the findings should be interpreted with caution, as different outcomes may emerge with more complex tasks or extensive practice, even when the task primarily relies on visual information.

### 4.1 Conclusion

The effects of self-control on motor learning have been extensively investigated; however, the specific effects of self-controlled feedback timing remain underexplored. Moreover, although the mechanisms by which self-control influences motor learning have been explored from the perspectives of motivation and information processing, a consensus has yet to be established. The findings of this study demonstrate the effectiveness of self-controlled feedback timing in motor learning during a grasping force control task in young adults and suggest that this effect may be mediated by the dynamic interplay between intrinsic motivation and information-processing. These findings highlight the importance of self-controlled feedback timing as a means of accommodating variability in task characteristics and learners’ skill level, thereby providing a flexible approach to optimizing motor learning. Future research should examine whether these effects generalize to other populations, tasks, and learning contexts.

## Data Availability

The raw data supporting the conclusions of this article will be made available by the authors, without undue reservation.
